# Intravitreal injection of adenosine A_2A_ receptor antagonist reduces neuroinflammation, vascular leakage and cell death in the retina of diabetic mice

**DOI:** 10.1038/s41598-019-53627-y

**Published:** 2019-11-20

**Authors:** Inês Dinis Aires, Maria Helena Madeira, Raquel Boia, Ana Catarina Rodrigues-Neves, Joana Margarida Martins, António Francisco Ambrósio, Ana Raquel Santiago

**Affiliations:** 1 Coimbra Institute for Clinical and Biomedical Research (iCBR), Faculty of Medicine, University of Coimbra, 3000-548 Coimbra, Portugal; 2 CNC.IBILI Consortium, University of Coimbra, Coimbra, Portugal; 3 Center for Innovative Biomedicine and Biotechnology (CIBB), University of Coimbra, Coimbra, Portugal

**Keywords:** Retinal diseases, Diabetes complications

## Abstract

Diabetic retinopathy is a major complication of diabetes mellitus and a leading cause of blindness. The pathogenesis of diabetic retinopathy is accompanied by chronic low-grade inflammation. Evidence shows that the blockade of adenosine A_2A_ receptors (A_2A_R) affords protection to the retina through the control of microglia-mediated neuroinflammation. Herein, we investigated the therapeutic potential of an antagonist of A_2A_R in a model of diabetic retinopathy. Type 1 diabetes was induced in 4–5 months old C57BL/6 J mice with a single intraperitoneal injection streptozotocin. Animals were treated one month after the onset of diabetes. The A_2A_R antagonist was delivered by intravitreal injection once a week for 4 weeks. Microglia reactivity and inflammatory mediators were increased in the retinas of diabetic animals. The treatment with the A_2A_R antagonist was able to control microglial reactivity and halt neuroinflammation. Furthermore, the A_2A_R antagonist rescued retinal vascular leakage, attenuated alterations in retinal thickness, decreased retinal cell death and the loss of retinal ganglion cells induced by diabetes. These results demonstrate that intravitreal injection of the A_2A_R antagonist controls inflammation, affords protection against cell loss and reduces vascular leakage associated with diabetes, which could be envisaged as a therapeutic approach for the early complications of diabetes in the retina.

## Introduction

Diabetic retinopathy is a major complication of diabetes mellitus and a leading cause of blindness and vision impairment in working-age adults^1^. Blood-retinal-barrier (BRB) breakdown is a hallmark of the disease^2^. The current available treatments mainly target neovascularization through the use of anti-vascular endothelial growth factor therapies, laser treatment and surgery^3^. Nevertheless, diabetic retinopathy is now considered a neuro-vascular disease in which a low-grade chronic inflammatory environment contribute to BRB breakdown and retinal dysfunction^1,4–8^. This inflammatory state is mainly due to glial cell dysfunction and BRB breakdown, possible induced by the death of retinal cells^4–7^. Microglial cells were shown to impact retinal cell function in diabetic conditions by increasing the pro-inflammatory milieu^9–14^. Taking the role of inflammation in the pathophysiology of diabetic retinopathy, therapeutic strategies targeting the control of neuroinflammation may offer protective options to manage diabetic retinopathy, halting disease progression.

Adenosine is a neuromodulator of the central nervous system exerting its actions through the activation of four adenosine receptors, A_1_R, A_2A_R, A_2B_R and A_3_R. Several reports demonstrate that detrimental microglial cell response might be amended by A_2A_R antagonists in different brain and retinal diseases^15–21^. In the brain, A_2A_R was found to be involved in the control of blood-brain barrier (BBB) permeability^22,23^. Therefore, we hypothesized that the blockade of A_2A_R might confer protection to the retina by modulating microglia reactivity, thus altering the course of the effects of diabetes. To address this hypothesis, we used an animal model of type 1 diabetes, which was treated with a selective A_2A_R antagonist, SCH 58261, by an intravitreal injection every week, for four weeks, starting one month after the onset of diabetes. This experimental protocol allows the study of the therapeutic potential of the A_2A_R antagonist, since the retina is already affected by the disease when the treatment is initiated.

## Results

### Retinal thickness is reduced one month after diabetes induction

All the animals used in this study were monitored for weight and glycemic before diabetes induction (baseline), and 1 and 2 months after the onset of diabetes. Hemoglobin A1C (HbA1C) was measured only in a subset of animals after 2 months of diabetes induction, randomly chosen. Biochemical data and weight of the animals from this sub-set are presented in Table 1.

**Table 1:** Representative data on glycemia, weight and HbA1c of the animals used in the study

	Control			Diabetic		
	Baseline	1 month	2 months	Baseline	1 month	2 months
Glycemia (mg/dL)	240 ± 10.5	238 ± 12.0	246 ± 3.4	244 ± 11.8	385 ± 28.8 ***	404 ± 29.0***
Weight (g)	26.5 ± 1.1	27.7 ± 1.0	27.6 ± 0.9	26.0 ± 0.9	25.9 ± 1.1	25.3 ± 0.8
HbA1c (%)	—	—	4.9 ± 0.08	—	—	7.0 ± 0.4
n	5–7			9–12		

***p < 0.001, compared with baseline, One-way ANOVA test, followed by Holm-Šídák multiple comparison test.

**Figure 1:**
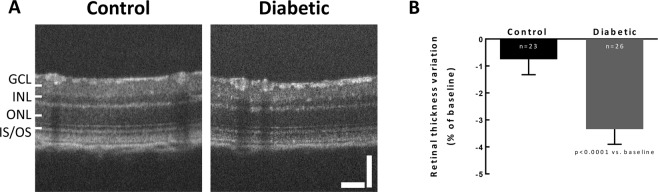
Retinal thickness is reduced one month after diabetes induction. (**A**) Representative OCT images. (**B**) OCT was performed before (baseline) and 4 weeks after diabetes induction, and retinal thickness was measured in OCT images after segmentation. The results were normalized to baseline, n = 23–26 animals. Wilcoxon matched-pairs signed rank test. GCL: ganglion cell layer; INL: inner nuclear layer; ONL: outer nuclear layer; IS-OS: photoreceptors inner and outer segments. Scale bars: 50 µm.

Retinal thinning has been reported as an early alteration in diabetic animals^24^. Four weeks after the onset of diabetes, retinal thickness significantly (p < 0.0001) decreased in diabetic animals when compared with control animals (Fig. 1), a structural indication of alterations due to diabetes. Since the aim of this work was to investigate the therapeutic potential of a selective A_2A_R antagonist for the treatment of diabetic retinopathy, we wanted to start the intravitreal injections after established retinal alterations. Therefore, the A_2A_R antagonist was injected intravitreally thereafter every week, in a total of 4 injections.

### Treatment with A_2A_R antagonist decreases microglia reactivity in the retina of diabetic mice

**Figure 2:**
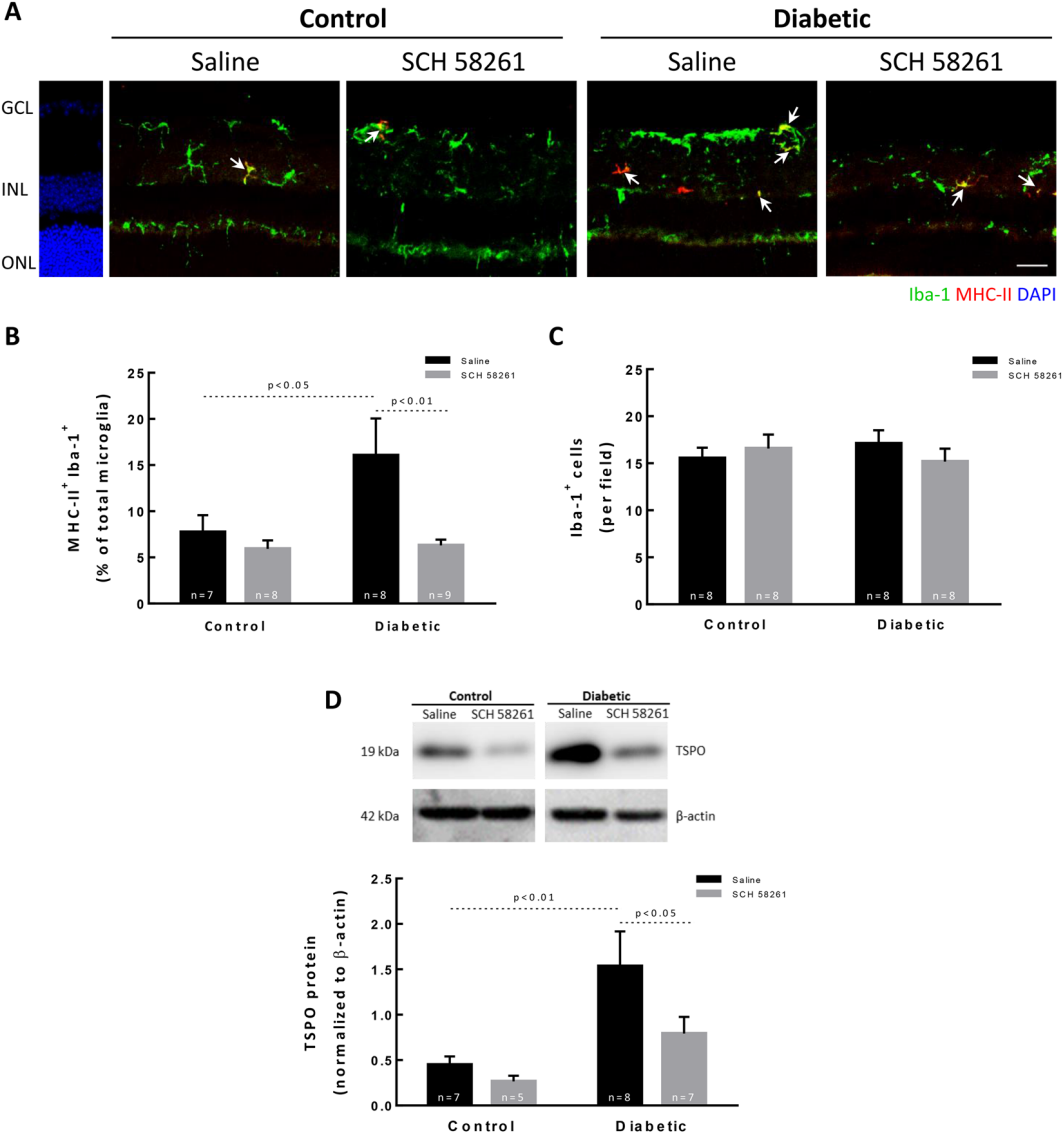
Treatment with A_2A_R antagonist decreases microglia reactivity in the retina of diabetic mice. (**A**) Retinal sections were stained with antibodies against Iba-1 (green) and MHC-II (red). Nuclei were stained with DAPI (blue). Representative images are depicted and arrows indicate some MHC-II^+^ Iba-1^+^ cells found in each condition. (**B**) Activated microglia (MHC-II^+^ Iba-1^+^ cells) were counted and normalized to the percentage of total microglial cells (Iba-1^+^ cells) from 7–9 animals. (**C**) The number of microglia per retinal section was counted. (**D**) TSPO protein levels were assessed by Western blot and the results are expressed as a ratio to β-actin from 6–9 independent experiments. Representative Western blots are presented. Full length uncropped images are presented as Supplementary Fig. 1. One-way ANOVA test, followed by Holm-Šídák multiple comparison test. GCL: ganglion cell layer; INL: inner nuclear layer; ONL: outer nuclear layer. Scale bar: 100 µm.

A large body of evidence demonstrate that inflammation contributes to diabetic retinopathy and microglia reactivity occurs early in the course of the disease^11^. Microglia were labelled with antibodies against ionized calcium-binding adaptor molecule 1 (Iba-1, a marker of microglia) and major histocompatibility complex class II (MHC-II, highly expressed in reactive microglia) (Fig. 2A). Therefore, microglial cells (Iba-1^+^ cells) labeled with MHC-II (MHC-II^+^ Iba-1^+^ cells) were considered reactive microglia^15^. As expected, microglia reactivity was increased in the retina of diabetic animals (16.1 ± 4.0% of total Iba-1^+^ cells; p < 0.05) when compared with the retinas of control animals (7.7 ± 1.8% of total Iba-1^+^ cells) (Fig. 2B). The intravitreal injection of SCH 58261, the A_2A_R antagonist, significantly decreased microglial reactivity in the retinas of diabetic animals (6.3 ± 0.6% of total Iba-1^+^ cells; p < 0.01). The administration of SCH 58261 to non-diabetic animals did not significantly change the number of reactive microglia (5.9 ± 0.9% of total Iba-1^+^ cells). These alterations in microglia reactivity in diabetic animals were not due to changes in the total number of microglia (Iba-1^+^ cells) (Fig. 2C). The Translocator protein (18 kDa) (TSPO) is constitutively expressed in retinal microglia and in inflammatory conditions TSPO expression increases^25,26^. Therefore, the effect of SCH 58261 on microglia reactivity was further studied by evaluating the protein levels of TSPO (Fig. 2D). Diabetes significantly increased by 3.4-fold the protein levels of TSPO in the retina of diabetic animals (p < 0.01) when compared with the control condition (saline-injected retinas from non-diabetic animals) (Fig. 2D), and this effect was abolished by the A_2A_R antagonist. The treatment of non-diabetic animals with the A_2A_R antagonist did not change TSPO protein levels (Fig. 2D).

Overall, these results indicate that the A_2A_R antagonist has the potential to control diabetes-induced retinal microglia reactivity.

### Treatment with A_2A_R antagonist impacts retinal neuroinflammation in the diabetic retinas

**Figure 3:**
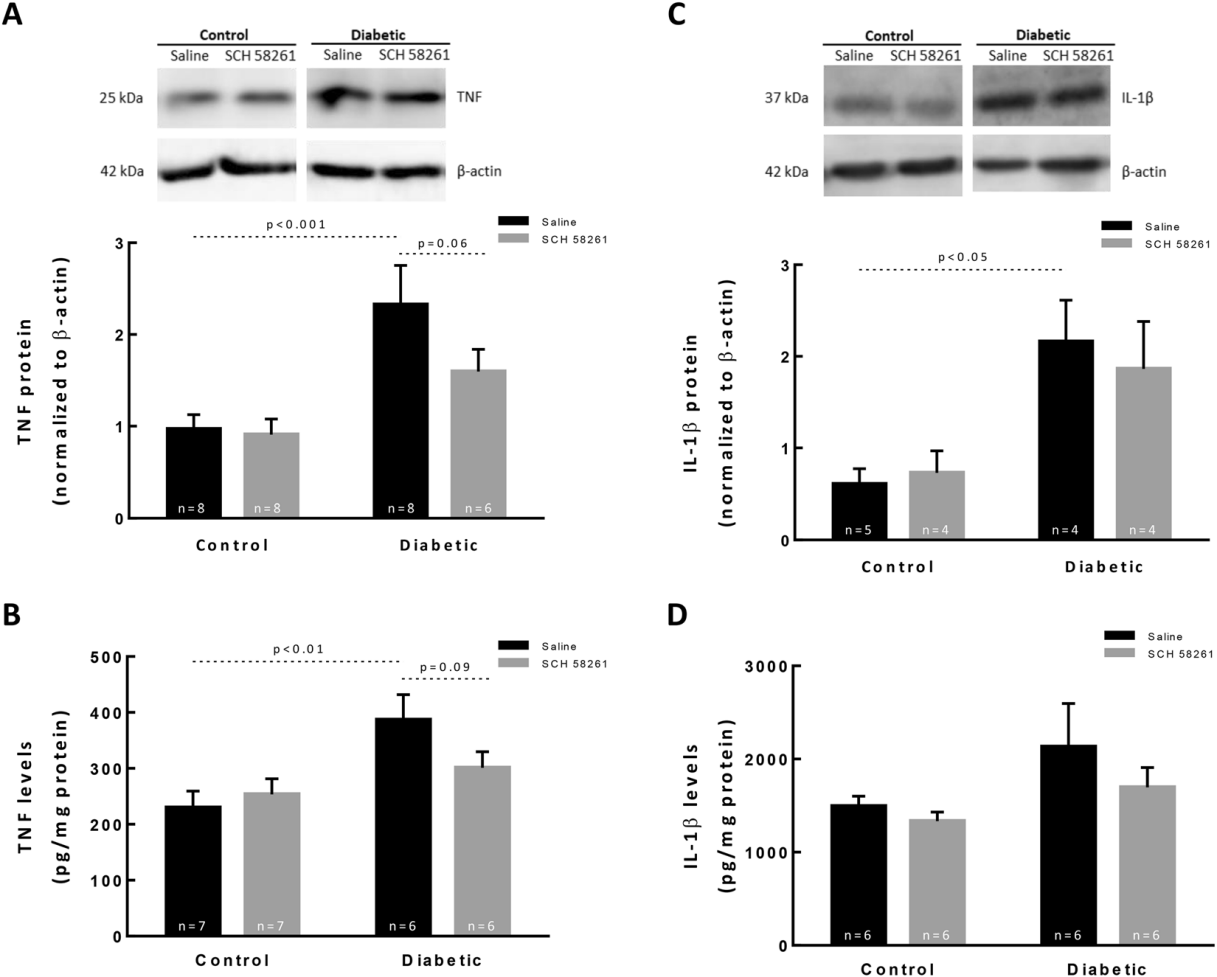
Treatment with A_2A_R antagonist impacts retinal neuroinflammation in the diabetic retinas. Retinal extracts were assayed for Western blot analysis of TNF (**A**) and IL-1β (**C**), and for ELISA to quantify TNF (**B**) and IL-1β (**D**). The results obtained from Western blot are expressed as the ratio to β-actin from 6–9 independent experiments. Representative Western blots are presented. Full length uncropped images are presented as Supplementary Fig. 2 (**A,C**). The results obtained by ELISA are presented as a ratio to the total amount of protein in the retina from 6–7 independent experiments for TNF, and from 4–5 independent experiments for IL-1β (**B,D**). One-way ANOVA test, followed by Holm-Šídák multiple comparison test.

The levels of the pro-inflammatory cytokines (tumor necrosis factor) TNF and (interleukin 1 beta) IL-1β in the retina were determined by Western blot and ELISA (Fig. 3). Several cytokines have been demonstrated to play a role in diabetic retinopathy^5,27^, including TNF and IL-1β. The A_2A_R antagonist was reported to decrease the levels of these two cytokines in other experimental models, including in the retina^16,18,28^. Therefore, in the current work we focused on assessing the effect of SCH 58261 on the levels of TNF and IL-1β in the retinas of diabetic animals, although we do not exclude the contribution of other cytokines to the disease progression. The protein expression of TNF was significantly increased in the diabetic retinas when compared with control retinas, as assessed by Western blot (Fig. 3A, p < 0.001). The TNF levels were also quantified by ELISA (Fig. 3B), and a significant increase in the retinas of diabetic animals was found (386.8 ± 110.2 pg/mg protein, p < 0.01), when compared with control retinas (229.9 ± 78.0 pg/mg protein). While the treatment of non-diabetic animals with A_2A_R antagonist did not modify the protein levels of TNF, the treatment of diabetic animals slightly reduced the protein levels of TNF. The IL-1β protein levels were also increased by 3.5-fold in the retinas of diabetic animals (p < 0.05) when compared with control retinas (Fig. 3C). Diabetes significantly increased the IL-1β levels in the retinas (2130.5 ± 1140.0 pg/mg protein; p < 0.001) when compared with the control (1492.9 ± 264.2 pg/mg protein) (Fig. 3D). The treatment of diabetic animals with A_2A_R antagonist did not modify the levels of IL-1β.

### Treatment with SCH 58261 decreases iNOS in the diabetic retinas

**Figure 4:**
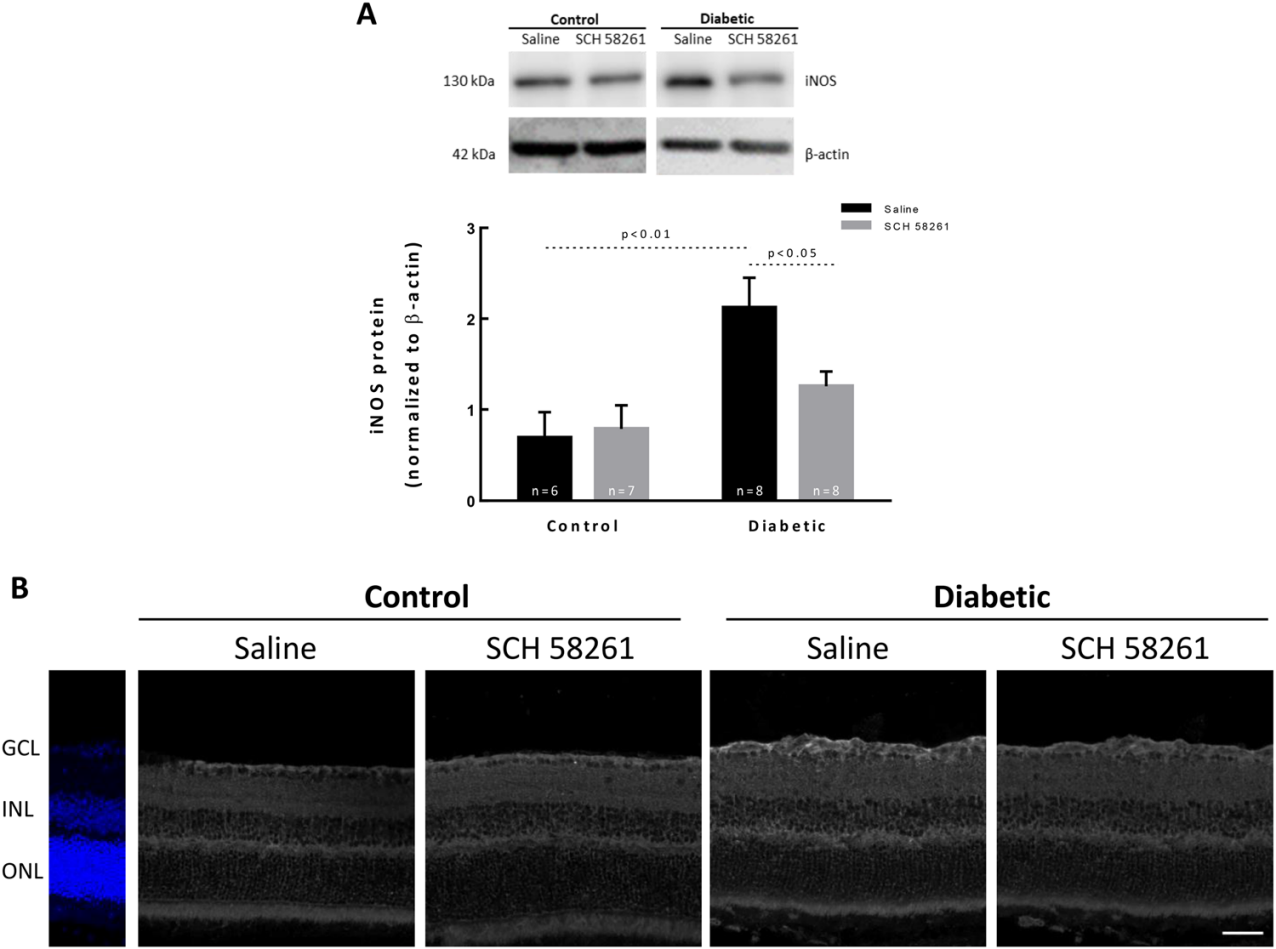
Treatment with SCH 58261 decreases iNOS in the diabetic retinas. (**A**) The protein levels of iNOS were evaluated by Western blot. The results are presented as a ratio to β-actin, from 6–8 independent experiments. Representative Western blots are presented. Full length uncropped images are presented as Supplementary Fig. 3. (**B**) Retinal sections were immunolabeled for iNOS (grey). Nuclei were stained with DAPI (blue). One-way ANOVA test, followed by Holm-Šídák multiple comparison test. GCL: ganglion cell layer; INL: inner nuclear layer; ONL: outer nuclear layer. Scale bar: 100 µm.

An increase in the protein levels of inducible nitric oxide synthase (iNOS) has been reported in the retina in diabetic conditions^29^. The protein levels of iNOS were significantly increased (3-fold change) in the retinas of diabetic animals (injected with saline) when compared with the control condition (p < 0.01, Fig. 4A). The treatment with the A_2A_R antagonist significantly decreased the iNOS protein levels (p < 0.05) comparatively to the diabetic retinas treated with saline. The intravitreal injection of SCH 58261 to non-diabetic animals did not change iNOS protein levels. Despite the overall increase in iNOS protein in the retinas of diabetic mice and the decrease by A_2A_R blockade (observed both by Western blot and by immunohistochemistry, Fig. 4), we were not able to identify a specific cell type responsible for these alterations since there were no changes in the distribution of iNOS in retinal vertical sections (Fig. 4B).

### Administration of the A_2A_R antagonist decreases cell death in the retina of diabetic animals

**Figure 5:**
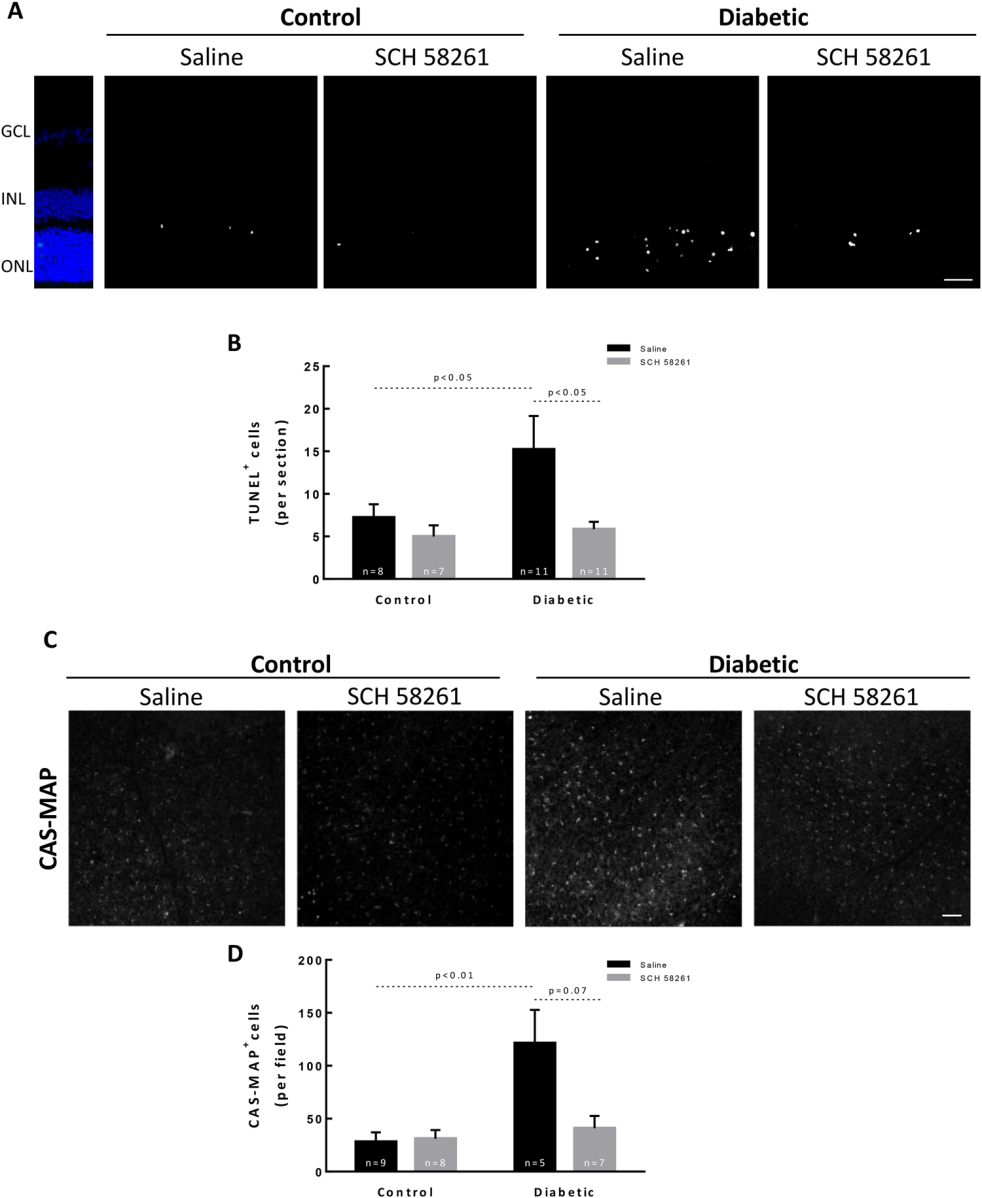
Administration of the A_2A_R antagonist decreases cell death in the retina of diabetic animals. (**A**) Cell death was evaluated by TUNEL assay in vertical retinal sections. Nuclei were stained with DAPI (blue). Representative images are depicted (**B**). The number of TUNEL^+^ cells (white) was counted from 7 to 11 independent experiments. (**C**) The presence of active caspases in the ganglion cell layer was assessed with CAS-MAP probe (grey). Representative images are depicted. (**D**) The number of cells CAS-MAP^+^ was counted from 5–9 independent experiments. One-way ANOVA test, followed by Holm-Šídák multiple comparison test. GCL: ganglion cell layer; INL: inner nuclear layer; ONL: outer nuclear layer. Scale bar: 100 µm.

Diabetes results in the death of retinal cells^30^. It is widely accepted that neuroinflammation triggers cell death, including in the diabetic retina^31^. Retinal cell death was evaluated by TUNEL assay in retinal sections (Fig. 5). The number of TUNEL^+^ cells significantly increased after 8 weeks of diabetes (15.2 ± 3.9 TUNEL^+^ cells per field, p < 0.05) when compared with the saline injected control retinas (7.2 ± 1.5 TUNEL^+^ cells per field) (Fig. 5A,B). The intravitreal injection of SCH 58261 to diabetic animals significantly decreased diabetes-induced retinal cell death (5.8 ± 0.9 TUNEL^+^ cells per field, p < 0.05).

The activation of caspase-dependent signaling apoptotic mechanisms has been associated with the loss of retinal cells in diabetic retinopathy, including retinal ganglion cells (RGCs)^32–34^. The presence of active caspases in the ganglion cell layer was evaluated using the CAS-MAP reagent, a probe to detect active caspases 1, 2, 3, 6, 8, 9, or 10 (Fig. 5C). Diabetes significantly increased the number of apoptotic cells in the ganglion cell layer, as indicated by the increase in the number of cells labeled with CAS-MAP (121.3 ± 31.5 CAS-MAP^+^ cells, p < 0.01) when compared with control retinas (28.2 ± 9.0 CAS-MAP^+^ cells) (Fig. 5C,D). The intravitreal injection of SCH 58261 to the retinas of diabetic animals attenuated the number of retinal cells undergoing caspase-dependent cell death (41.0 ± 11.7 CAS-MAP^+^ cells, p = 0.07) (Fig. 5C,D). The administration of SCH 58261 to non-diabetic animals did not change the number of retinal cells with active caspases (31.1 ± 8.3 CAS-MAP^+^ cells).

### A_2A_R antagonist attenuates RGC loss in a mouse model of diabetic retinopathy

**Figure 6:**
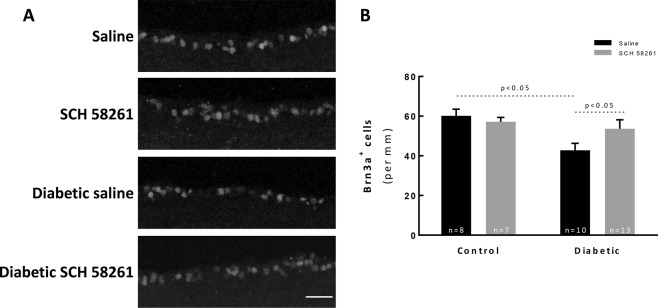
A_2A_R antagonist attenuates RGC loss in a mouse model of diabetic retinopathy. (**A**) The number of RGCs was evaluated in vertical sections by immunolabeling against Brn3a (grey). The nuclei were stained with DAPI (blue). Representative images are depicted. (**B**) The number of Brn3a^+^ cells were counted, and the results were normalized to the length of the section; from 7–13 animals. One-way ANOVA test, followed by Holm-Šídák multiple comparison test. GCL: ganglion cell layer; INL: inner nuclear layer; ONL: outer nuclear layer. Scale bar: 100 µm.

Previous findings in the STZ-induced diabetic mouse model have reported loss of RGCs^30,35^. RGCs were labeled with an antibody anti-Brn3a (Fig. 6), a transcription factor that is expressed by these cells^36^. There was a decrease in the number of RGCs after 8 weeks of diabetes (42.8 ± 3.5 Brn3a^+^ cells/mm, p < 0.05) when comparing with control animals (60.2 ± 3.3 Brn3a^+^ cells/mm, Fig. 6A,B). The A_2A_R antagonist did not change the number of Brn3a^+^ cells of non-diabetic animals (57.2 ± 2.2 Brn3a^+^ cells per mm). However, the treatment of diabetic animals with the A_2A_R antagonist promoted the survival of RGCs (53.6 ± 4.5 Brn3a^+^ cells/mm, p < 0.05).

### Intravitreal administration of SCH 58261 inhibits retinal thinning in diabetic animals

**Figure 7:**
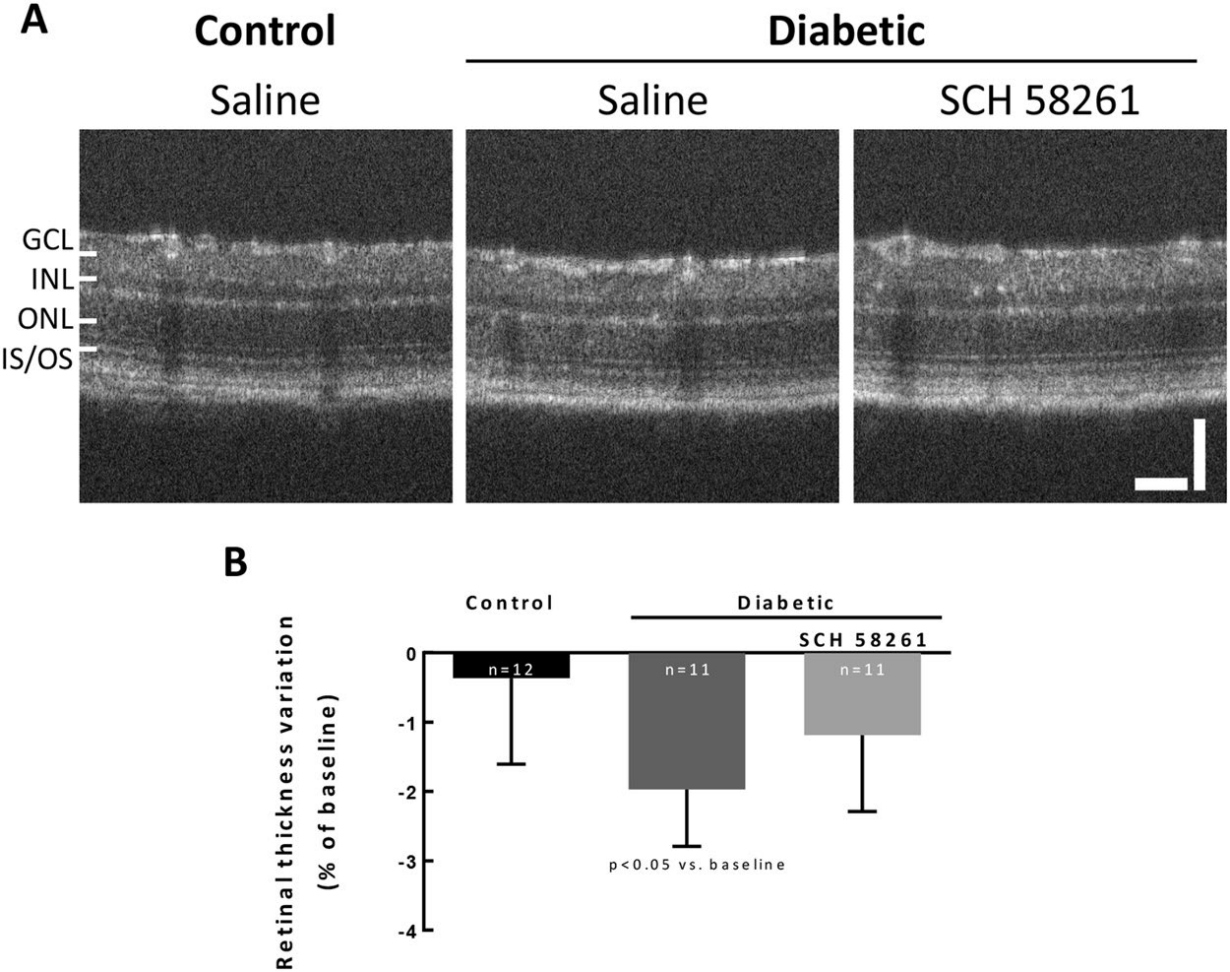
Intravitreal administration of SCH 58261 inhibits retinal thinning in diabetic animals. Retinal thickness was calculated after layer segmentation in OCT images. (**A**) Representative OCT images at eight weeks of diabetes. (**B**) The results were normalized to baseline. Wilcoxon matched-pairs signed rank test. GCL: ganglion cell layer; INL: inner nuclear layer; ONL: outer nuclear layer; IS-OS: photoreceptor inner and outer segments. Scale bars: 50 µm.

Retinal thinning can be a feature of retinal cell loss associated with diabetic retinopathy progression^37^. Retinal thickness was measured by OCT. Since OCT allows for longitudinal studies, retinal thickness was referred to the baseline and alterations to the baseline were documented (Fig. 7). The thickness of the retinas of control animals remained quite constant throughout the study. However, thinning was found in the retinas of diabetic (8 weeks) animals (p < 0.05 when compared with the baseline; Fig. 7A,B). The treatment of diabetic animals with SCH 58261 for 4 weeks did not statistically modify retinal thickness, when compared with the baseline values (Fig. 7A,B), which might be in accordance with a protective effect of the A_2A_R antagonist.

### Treatment with A_2A_R antagonist reduces retinal vascular leakage in diabetic mice

**Figure 8:**
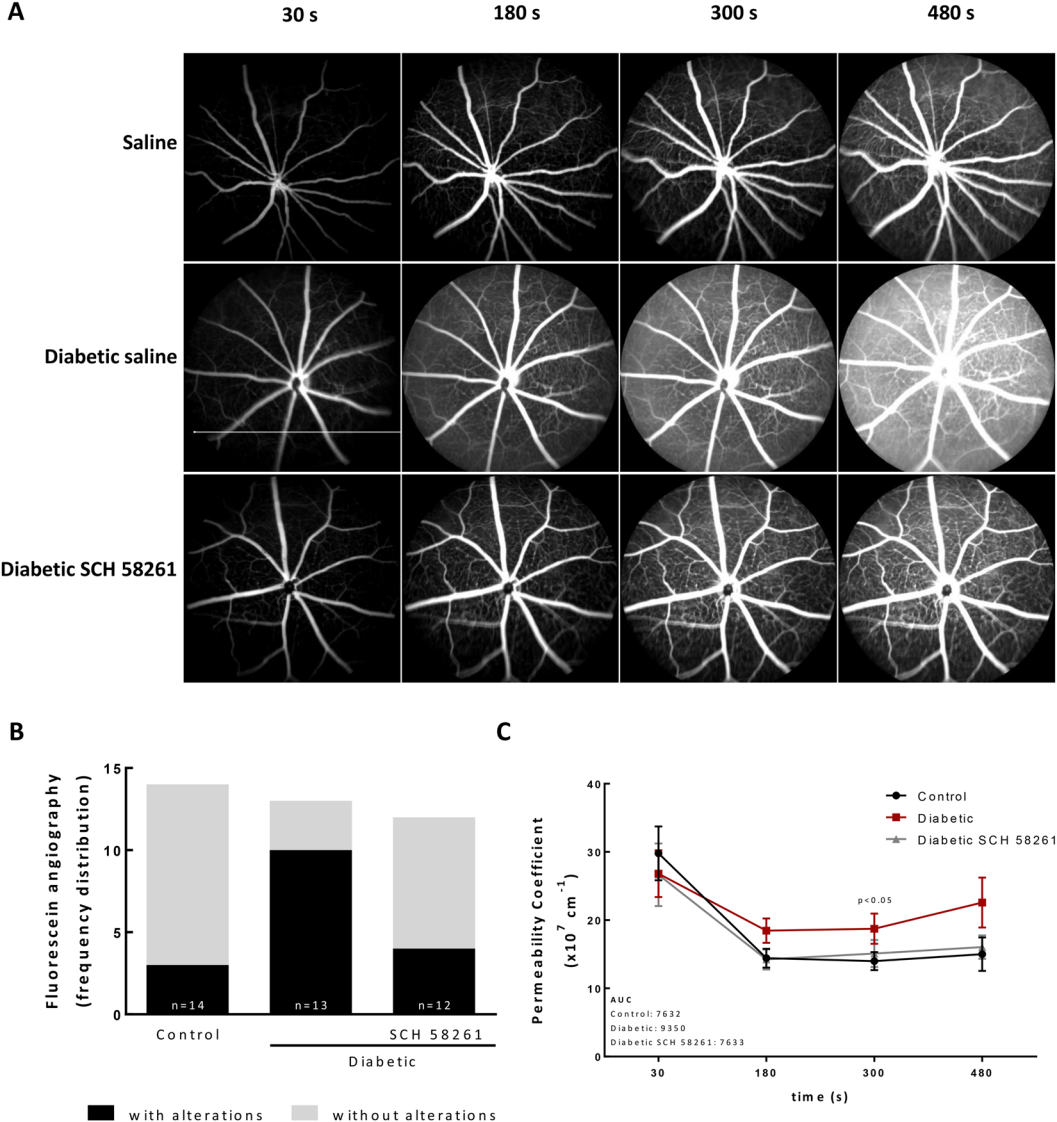
Treatment with A_2A_R antagonist reduces retinal vascular leakage in diabetic mice. (**A**) Fluorescein angiography was performed after intraperitoneal injection of 5% fluorescein in control and diabetic non-treated animals or treated with the A_2A_R antagonist. Images were acquired with the Micron IV (Phoenix Technology) in equivalent time frames. The images represent the distribution of fluorescein across the retina, acquired 30 seconds, 180 seconds, 300 seconds and 480 seconds after injection. (**B**) The images were classified for the presence or absence of extravasation of fluorescein by 3 independent researchers and the frequency of retinas with and without fluorescein leakage was expressed in percentage of total animals. (**C**) Permeability coefficient obtained by the quantification of the fluorescence between a major vessel and the interstitial space over time. The area under the curve (AUC) of the permeability coefficient is presented in the graph insert for the different experimental conditions. Permeability coefficient was analyzed with two-way ANOVA using Fisher’s LSD test.

BRB dysfunction is a hallmark of diabetic retinopathy and can be assessed by fluorescein angiography. Vascular leakage, as a measurement of BRB dysfunction, was assessed *in vivo* by fluorescein angiography (Fig. 8). In most of non-diabetic mice (79% of animals), fluorescein was maintained within the retinal vessels, indicating absence of vascular leakage. The angiograms of diabetic animals showed regions of vascular leakage and an overall diffuse green haze, indicating extravascular fluorescein, a sign of vascular leakage. The treatment of diabetic animals with the A_2A_R antagonist reduced the number of animals with retinal vascular fluorescein leakage (Fig. 8A,B), suggesting that the A_2A_R antagonist restored the BRB integrity lost in the course of diabetes. Furthermore, the analysis of the permeability coefficient, obtained from the images of the angiograms over time, shows a tendency to an increase in permeability in the retina of diabetic animals, which was not present in the eyes of the diabetic animals treated with the A_2A_R antagonist (Fig. 8C). Indeed, the curve profile of the diabetic eyes treated with SCH 58261 was very similar to the control group (Fig. 8C). The area under the curve (AUC) (Fig. 8C), for each group suggested that the treatment with A_2A_R antagonist may attenuate the effects of diabetes on vascular leakage. Moreover, the permeability coefficient of the diabetic animals was significantly higher than the control animals and SCH 58261 was able to significantly decrease the permeability coefficient of diabetic animals (at 300 seconds, two-way ANOVA using Fisher’s LSD test, p < 0.05).

## Discussion

In this work, we demonstrate the therapeutic potential of an A_2A_R antagonist for the treatment of the retinal complications in diabetes. Importantly, the treatment of STZ-induced type I diabetic mice with the A_2A_R antagonist by intravitreal injection was initiated 4 weeks after the onset of diabetes, when the retina is already compromised^6,29,38^.

Diabetes and elevated glucose concentration increase the expression of A_2A_R in retinal cell cultures and in animal models of diabetes^39,40^. Retinal microglia are endowed with A_2A_R^16,28,41^, but other cells in the retina may also express the receptor (Supplementary Fig. 4 and^42^). We and others demonstrated that the blockade of A_2A_R affords protection to the retina and brain against noxious conditions involving microglia-mediated neuroinflammation control^15–20,28,43,44^. The intravitreal administration of SCH 58261 in diabetic animals decreased retinal microglial reactivity. Previously, we demonstrated that blocking A_2A_R in microglia prevents the reactivity of these cells and retinal neural cell loss^28^, suggesting that the effects observed herein occur through the modulation of microglia phenotype. One limitation of this study is the use of only one drug to infer on the therapeutic potential of A_2A_R for the treatment of diabetic retinopathy. In previous works, we demonstrated the beneficial effects of oral administration of KW6002 (selective A_2A_R antagonist) and caffeine (non-selective adenosine receptor antagonist) to the retina, through the control of microglia-mediated neuroinflammation^15,45^. Since caffeine is a non-selective adenosine receptor antagonist it would not be helpful when elucidating the role of A_2A_R in the diabetic retina. Moreover, the oral administration of KW6002 or caffeine, as performed in past works, could interfere with other signaling pathways given the systemic nature of diabetes. Another issue is the lack of the pharmacological evidence of the role played by A_2A_R, using the A_2A_R selective agonist. These issues are critically important since the effects mediated by A_2A_R are bidirectional and depend on the target cell^46^. The global A_2A_R knockout mouse would not be relevant to study the therapeutic potential of A_2A_R blockade since this receptor interferes with the development of retinal vasculature, neuronal wiring and glial cell function, and impacts visual function^47–50^. Others demonstrated that the systemic delivery of an A_2A_R agonist decreases retinal cell death in diabetic animals^41^. The apparent contradictory findings might be explained by the different routes of drug administration, doses used and stage of the disease.

Diabetic retinopathy is a low-grade chronic inflammatory disease and a large body of evidence supports the role of inflammation in BRB dysfunction^1,4,51,52^. Diabetes increased the retinal levels of TNF and IL-1β, but the treatment with SCH 58261 only slightly attenuated the expression of TNF. The differential modulation of the levels of proinflammatory cytokines by the A_2A_R antagonist might be dependent on the pathological conditions or the animal species being evaluated. In the current work, the treatment with the A_2A_R antagonist was initiated four weeks after the onset of diabetes. This may explain the lack of effects of SCH 58261 on reducing the levels of TNF and IL-1β since at four weeks of diabetes the inflammatory environment is already established^29^. In a previous work, we reported that a single intravitreal injection of SCH 58261 prevents the increase in the expression of IL-1β induced by transient retinal ischemia in Wistar rats, without altering the levels of TNF^16^. In contrary, the mouse model of transient retinal ischemia promotes an increase in TNF without interfering with IL-1β retinal levels^15^.

Also, microglial reactivity and neuroinflammation were shown to contribute to retinal degeneration in diabetic retinopathy^9–13^. Caspases are family of cysteine proteases known to be involved in the initiation and execution of apoptosis and have been implicated in the death of RGCs^34^, including in diabetic retinopathy^32,53^. Our results show an increase in the number of cells with active caspases within the GCL. The GCL is composed not only by RGCs but also astrocytes and displaced amacrine cells, and it is estimated that in the rodent retina RGCs comprise 50% of the total GCL population^54,55^. We found a reduction in the number of RGCs, suggesting that diabetes is causing caspase-dependent RGC death. Nevertheless, displaced amacrine cells may also degenerate in diabetes^56^. Moreover, we do not rule out the possibility that active caspases are also present in other retinal layers, since other cells, like photoreceptors are also affected by diabetes^57–60^. Interestingly, TUNEL staining was mainly observed in the ONL. Several mechanisms orchestrate cell death in the retina during diabetes, and cell death does not occur at the same time window. The absence of TUNEL staining in the GCL may be due to the efficient phagocytosis by microglial cells that become reactive in the course of diabetes^61^ and actively phagocyte RGC debris^62^. The A_2A_R antagonist afforded protection to the retinas of diabetic animals, namely to RGCs, probably the controlling microglia reactivity, as demonstrated in other noxious conditions^15,16,18,28,63^.

The protein levels of iNOS were increased in the retinas of diabetic animals, as reported previously^29,64,65^. Once expressed, iNOS is constantly active thus producing high amounts of NO^66^, with detrimental effects to the retina, such as increased leukostasis and BRB breakdown^29^. The cells expressing iNOS were not identified but one can speculate, based on previous reports, that iNOS expression occurs mainly in microglia and astrocytes in the retina of diabetic mice^13,67^. In fact, the A_2A_R antagonist prevents iNOS increase in microglia triggered by noxious inflammatory conditions^16,18^. In the current work the treatment with the A_2A_R antagonist reduced the diabetes-induced expression of iNOS. Taking the role of iNOS to the disruption of BRB in mouse models of diabetic retinopathy^29,64^ and our data on the control of microglia-mediated neuroinflammation by A_2A_R antagonist, we might postulate that the blockade of the A_2A_R improves BRB properties by decreasing microglia reactivity. Nevertheless, it was demonstrated that A_2A_R activation mediates pathological angiogenesis in the retina by promoting HIF-1α accumulation and increased glycolysis that lead to endothelial cell proliferation and sprouting^68^, and therefore we cannot discard that A_2A_R antagonist may also be targeting endothelial cells.

Our data support the therapeutic potential of A_2A_R antagonists for the treatment of diabetic retinopathy. We can suggest the control of microglia-mediated neuroinflammation as the axis that will contribute to a decrease in proinflammatory mediators and nitrosative stress, maintenance of BRB integrity and neuroprotection. The A_2A_R emerges as a candidate to be further tested in the context of diabetic retinopathy.

## Materials and Methods

### Animals

C57BL/6 J mice were housed in a controlled environment (21.8 ± 0.1 °C and 67.6 ± 1.6% relative humidity, 12 hours light/12 hours dark cycle) with free access to water and standard rodent chow. All procedures were approved by the Animal Welfare Committee of the Faculty of Medicine of University of Coimbra (ORBEA: 24–2015) and conducted in accordance with the European Community directive guidelines for the use of animals in laboratory (2010/63/EU) transposed to the Portuguese law (Decreto-Lei 113/2013) and were also in agreement with the Association for Research in Vision and Ophthalmology statement for animal use.

### Induction of type 1 diabetes

Four-month-old C57BL/6 J mice were randomly assigned to control or diabetic animals. Diabetes was induced with streptozotocin (STZ; 150 mg/kg, i.p.) (Sigma-Aldrich, St Louis, MO, USA) prepared immediately before injection in citrate buffer (10 mM Na-citrate, pH 4.5) (Sigma-Aldrich, St Louis, MO, USA). Diabetes was confirmed one week later by tail blood glycemic values above 250 mg/dl. STZ-injected animals with glucose levels below 250 mg/dl were excluded from the study.

### Drug administration

Four weeks after the onset of diabetes, animals were treated with the A_2A_R selective antagonist (SCH 58261) or with the vehicle, by intravitreal injection (one injection per week, for four weeks). Animals were anesthetized with 2.5% isoflurane (IsoFlo; Abbott Laboratories, Chicago, USA) in 1 l/min O_2_, pupils were dilated (Tropicil Top®, 10 mg/ml) and topical anesthesia (oxybuprocaine hydrochloride, Anestocil®, 4 mg/ml) was applied. SCH 58261 (Tocris, Bristol, UK), 2 µl of 100 mM solution prepared in 0.9% NaCl, or 2 µl vehicle (0.9% NaCl) was injected into the vitreous using a 36 G needle connected to an intraocular injection kit (NanoFil™ Application Kits, World Precision Instruments, Hertfordshire, UK) coupled to a 10 µl syringe and an automated pump controlled with a footswitch (Micro4; World Precision Instruments, Hertfordshire, UK).

### Hemoglobin A1c measurement

At the end of the study, blood from the tail vein was collected and hemoglobin A1c (HbA1c) values were determined using a DCA Vantage analyzer according to the manufacturer instructions (Siemens, Munich, Germany). HbA1c was reported as percentage of the total albumin in each sample: % HbA1c = (HbA1c/Total Hemoglobin) × 100.

### Retinal cryosections

Animals were deeply anesthetized with ketamine (80 mg/kg; Imalgene® 1000) and xylazine (5 mg/kg; Ronpum® 2%) and transcardially perfused with phosphate-buffered saline (PBS; in mM: 137 NaCl, 2.7 KCl, 10 Na_2_HPO_4_, and 1.8 KH_2_PO_4_; pH 7.4) followed by 4% (w/v) paraformaldehyde (PFA). Eye cups were prepared as previously described^15^. Retinal cryosections of 14 µm thickness were cut in a cryostat (Leica CM3050 S, Leica Biosystems, Wetzlar, Germany) and mounted on Superfrost Plus glass slides (Menzel-Gläser; Thermo Scientific, Massachusetts, USA).

### Immunohistochemistry

Retinal sections were immunolabeled for Iba-1 and MHC-II as previously described^15^ using the antibodies listed in Table 2. Retinal sections were imaged using an inverted fluorescence microscope (Zeiss Axio HXP-120, Zeiss, Oberkochen, Germany) with a 20x objective (Plan Achromat 20 × /0.8 M27). The number of cells immunoreactive to Iba-1 was counted and the number of cells immunoreactive to both Iba-1 and MHC-II (reactive microglia) was normalized to the % of total microglia.

**Table 2:** List of primary and secondary antibodies used in this work.

	Supplier, Cat #	Host	Dilution	Technique
**Primary antibodies**				
anti-Iba-1	Wako, 019–19741	rabbit	(1:1000)	Microscopy
anti-MHC-II	eBioscience, 14–5321–82	rat	(1:500)	
anti-Brn3a	Santa Cruz, sc-31984	goat	(1:500)	
anti-β-actin	Sicgen, AB0041-500	goat	(1:1000)	Western blot
anti-TNF	Abcam, ab66579	rabbit	(1:1000)	
anti-iNOS	Santa Cruz, sc-650	rabbit	(1:500)	
anti-IL-1β	R&D Systems, AF-401-NA	goat	(1:1000)	
anti-PBR (TSPO)	Abcam, ab109497	rabbit	(1:1000)	
**Secondary antibodies**				
Alexa Fluor® 488 Goat Anti-Rabbit IgG (H + L)	Invitrogen, A11008	goat	(1:500)	Microscopy
Alexa Fluor® 568 Goat Anti-Rat IgG (H + L)	Invitrogen, A11077	goat	(1:500)	
Alexa Flour® 568 Donkey Anti-Goat (H + L)	Invitrogen, A11057	donkey	(1:500)	
anti-rabbit IgG (H + L), HRP	BioRad,1706515	goat	(1:10000)	Western blot
HRP-coupled anti-goat IgG (H + L)	Invitrogen, LTI 611620	rabbit	(1:10000)	
AP-coupled anti-goat IgG (H + L)	Invitrogen, LTI 611622	rabbit	(1:10000)	

### Terminal deoxynucleotidyl transferase (TdT)-mediated dUTP nick end labeling (TUNEL) assay

Cell death was determined in retinal sections by DeadEnd™ Fluorometric TUNEL assay following the manufacturer’s instructions (Promega, Madison, WI, USA). Nuclei were counterstained with 4′,6-diamidine-2′-phenylindole dihydrochloride (DAPI) (Invitrogen, Carlsbad, CA, USA) (1:2000). The preparations were mounted with Glycergel mounting medium (DAKO, Agilent, Santa Clara, CA, USA) and were observed in an inverted fluorescence microscope (Zeiss Axio HXP-120, Zeiss, Oberkochen, DE) with a 20x objective (Plan Achromat 20×/0.8 M27). The number of TUNEL-positive cells was counted in the entire retinal section (4 sections per eye).

### Caspase activity detection assay by *in vivo* labelling

The detection of active caspases within the GCL was performed using the pan-caspase CAS-MAP probe. Animals were anesthetized with 2.5% isoflurane (IsoFlo; Abbott Laboratories, Chicago, IL, USA) in 1 l/min O_2_. Following pupil dilation (Tropicil Top®, 10 mg/ml) and topical anesthesia (oxybuprocaine hydrochloride, Anestocil®, 4 mg/ml), 2 µl of 100 nM/kg of CAS-MAP probe (Vergence Bioscience, Minneapolis, MN, USA) was administered into the vitreous with a 36 G needle coupled to a Hamilton syringe. Then, 24 hours later, the animals were anesthetized with ketamine (80 mg/kg; Imalgene® 1000) and xylazine (5 mg/kg; Ronpum® 2%) and transcardially perfused with PBS and 4% (w/v) PFA. The retinas were then dissected and washed with PBS. Nuclei were stained with DAPI and the retinas were mounted with Glycergel mounting media (DAKO, Agilent, CA, USA). The samples were observed in a confocal microscope (LSM 710, Zeiss, Oberkochen, Germany) using a 20x objective (Plan-Apochromat 20 × /0.8 M27), and from each retina two images per quadrant were randomly acquired in the ganglion cell layer focusing plane. The number of CAS-MAP^+^ cells was counted per image.

### Western blot

Preparation of retinal extracts and Western blot was performed as previously described^69^. In order to avoid biased matched comparisons, samples from both retinas of each animal were loaded, side by side in the gel, assuming separated membranes for control and diabetic animals. Nevertheless, membranes were developed at the same time and comparisons were made to the β-actin ratio. The membranes were incubated with the antibodies described in Table 2. Immunolabeling was detected using WesternBright Sirius™ (Advansta, Menlo Park, CA, USA) or with ECF™ (GE Healthcare Amersham™, Little Chalfont, UK), in accordance with the manufacturer’s instructions.

### Enzyme-linked immunosorbent assay (ELISA)

The retinas were collected in ice-cold ELISA buffer (20 mM imidazole-HCl, 100 mM KCl, 1 mM MgCl_2_, 1 mM EGTA, 1 mM EDTA, 1% Triton X-100), supplemented with phosphatase inhibitors (10 mM NaF and 1 mM Na_3_VO_4_) and complete mini protease inhibitor cocktail tablets (Roche, Sigma-Aldrich, St Louis, MO, USA). The tissue was homogenized by sonication, centrifuged at 10000 × g for 5 min at 4 °C and the supernatant was collected. Protein concentration was determined using BCA (Pierce Biotechnology, Rockford, IL, USA). The levels of TNF and IL-β were quantified in accordance to the manufacturer’s instructions (PeproTech, London, UK), using a microplate reader (Synergy HT; Biotek, Winooski, VT, USA). The results were normalized to the total amount of protein in each sample.

### Optical coherence tomography (OCT)

Retinal thickness and structure were analyzed followed Spectrum Domain-OCT (coupled to a Phoenix Micron IV Retinal Imaging Microscope, Phoenix Technology, Pleasanton, CA, USA), as previously described^70^, with minor modifications as follows. Briefly, the animals were anesthetized using ketamine (80 mg/kg; Imalgene® 1000) and xylazine (5 mg/kg; Ronpum® 2%), the pupils were dilated using tropicamide (Tropicil Top®, 10 mg/ml) and oxybuprocaine (Anestocil®) was applied in the cornea for topical anesthesia. Corneal hydration was maintained using carmellose sodium (Celluvisc®). OCT was performed at baseline (before diabetes induction), one month after the onset of diabetes and at the end of the study (2 months after diabetes onset). Eye fundus images were obtained using the Micron IV Retinal Imaging Microscope for image guidance. The thickness of retinal layers was analyzed after OCT scan data segmentation with InSight (Phoenix Technology). For each eye, 4 B-scans were analyzed (2 images above and 2 images bellow the optic nerve). The results were normalized to the baseline, as follows:

$$\frac{x-xi}{x}\times 100 \% $$

x_i_ represents baseline retinal thickness; x represents retinal thickness at the defined timepoint.

### Fluorescein angiography

The animals were anesthetized as described for OCT and the eyes were treated equally. Animals were injected with 5% fluorescein (100 μl, i.p.), as described previously^71,72^. Fluorescein angiography images were acquired with the Micron IV Retinal Imaging Microscope (Phoenix Technology, Pleasanton, CA, USA) sequentially until saturation. For this experiment, only one eye per animal was analyzed (SCH 58261 treated and non-treated diabetic retinas are from different animals) in order to keep the acquisition time equivalents between conditions. The images were classified to the presence of leaky areas by three independent researchers that were masked to the experimental condition.

The permeability coefficient was estimated as previously described^73,74^, as follows. The fluorescence intensity of a major vessel and of the interstitial space was determined with ImageJ for 4 time points (30 seconds, 180 seconds, 300 seconds and 480 seconds after injection).

Fluorescence intensity ratio *F* was calculated to obtain the slope of the linear equation:

$$F=\varDelta C/t$$

*F* represents the fluorescence intensity in a given time point; *ΔC* represents the fluorescence difference between a major vessel and the interstitial space; *t* is the time after injection in seconds.

The permeability coefficient *P* was calculated applying the formula:

$$P=slopeF/(\Delta {\rm{C}}\times A)$$

*slope F* represents the slope obtained from the fluorescence intensity over time; *ΔC* represents the fluorescence difference between the major vessel and the interstitial space at a given time; *A* represents the area of the region of interest.

### Statistical analysis

Data is presented as mean ± SEM. The results were analyzed using Graphpad Prism software version 6.01 for Windows. The OCT data was analyzed using IBM SPSS statistics software version 24 for Windows. The distribution of the data was evaluated by Shapiro-Wilk normality test. The data with normal distribution was analyzed with parametric one-way ANOVA followed by Sidak’s multiple comparisons test, and for data without Gaussian distribution the statistical significance was determined with Kruskall-Wallis test followed by Dunn’s multiple comparison test or Mann-Whitney test, as indicated in the figure legends. For the analysis of OCT data, the Wilcoxon matched-pairs signed rank test was used. Fluorescein angiography was analyzed by two-way ANOVA using Fisher’s LSD test and the area under the curve (AUC) of the permeability coefficient was calculated setting the baseline at Y = 0. Statistical significance was defined for p < 0.05.

## Supplementary information

Supplementary Figures
