# Supplementary material for: Intravitreal injection of adenosine A2A receptor antagonist reduces neuroinflammation, vascular leakage and cell death in the retina of diabetic mice
Source: Sci Rep. 2019 Nov 20;9:17207. doi: 10.1038/s41598-019-53627-y (PMC6868354; doi:10.1038/s41598-019-53627-y)
Supplement: Supplementary file 1 — Supplementary Figures [file 41598_2019_53627_MOESM1_ESM.docx]

**Intravitreal injection of adenosine A_2A_ receptor antagonist reduces neuroinflammation, vascular leakage and cell death in the retina of diabetic mice**

Inês Dinis Aires^1,2,3^, Maria Helena Madeira^1,2,3^, Raquel Boia^1,2,3^, Ana Catarina Rodrigues-Neves^1,2,3^, Joana Margarida Martins^1,2,3^, António Francisco Ambrósio^1,2,3^, Ana Raquel Santiago^1,2,3^*

^1^Coimbra Institute for Clinical and Biomedical Research (iCBR), Faculty of Medicine, University of Coimbra, 3000-548 Coimbra, Portugal

^2^CNC.IBILI Consortium, University of Coimbra, Portugal

^3^Center for Innovative Biomedicine and Biotechnology (CIBB), University of Coimbra, Portugal

*Corresponding [asantiago@fmed.uc.pt](mailto:asantiago@fmed.uc.pt)


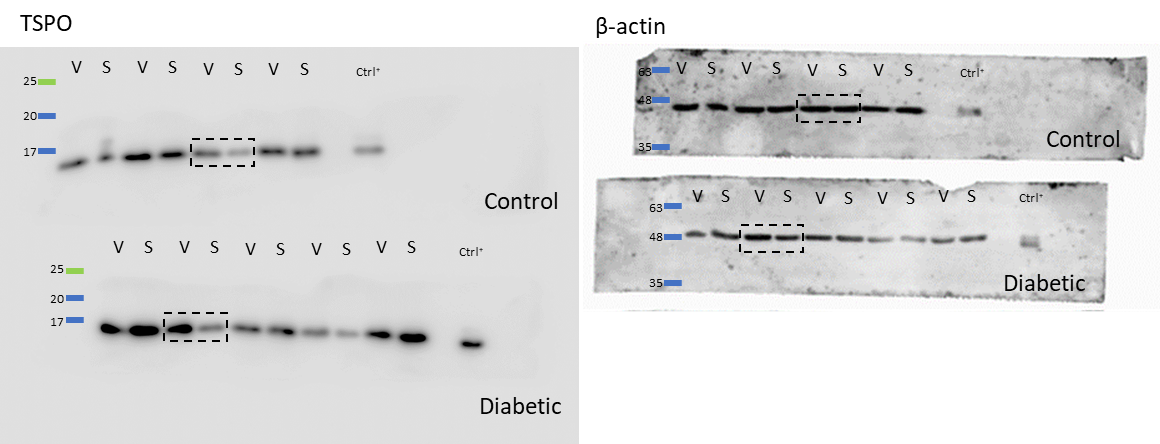


**Supplementary Figure 1 - Full length western blots from cropped images depicted in figure 2.** Samples from control or diabetic animals treated with the A_2A_R selective antagonist (S) or with the vehicle (V). Retinas from animals intravitreal injected with 2 µl of 1 mg/ml of lipopolysaccharide (LPS) were used as positive control.

**
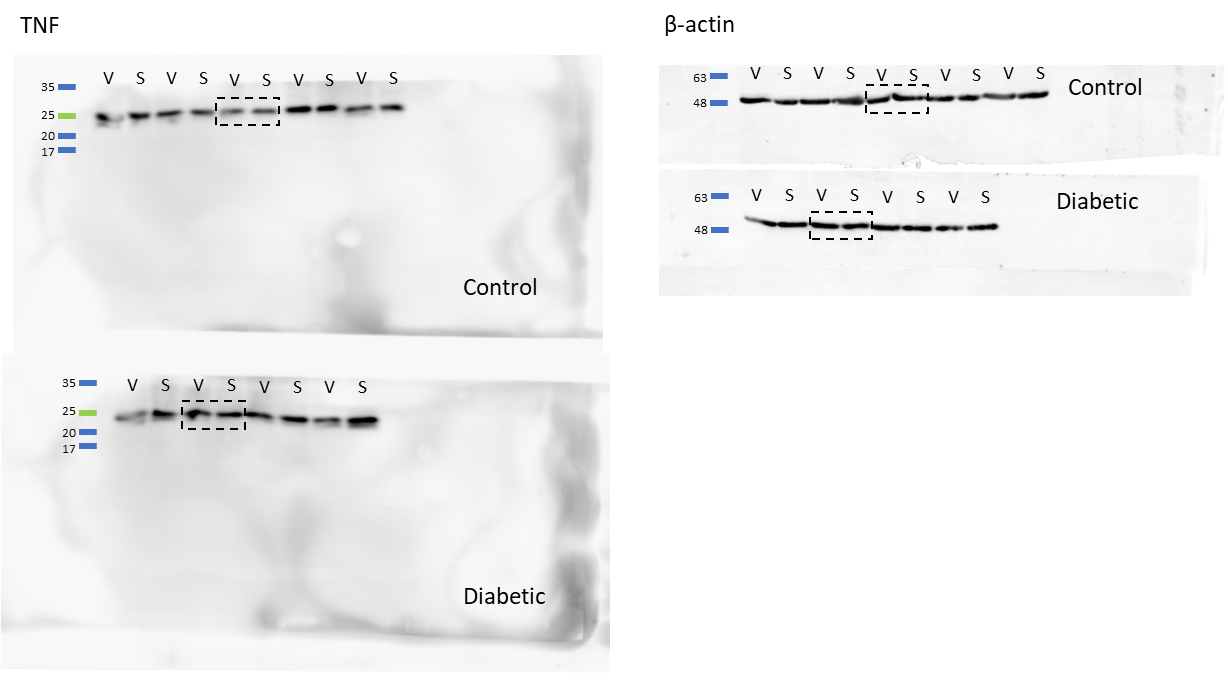
**

**
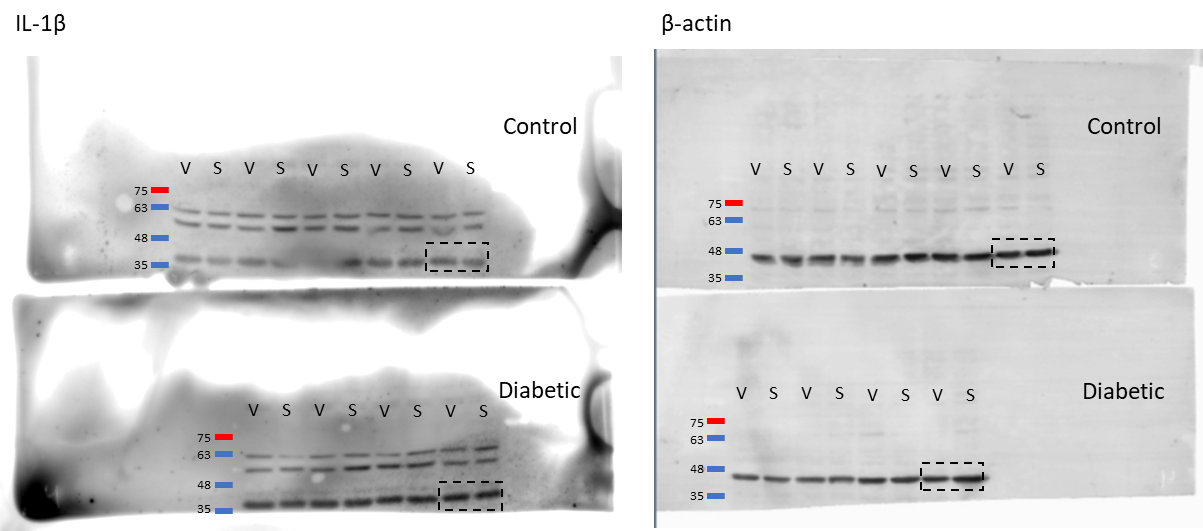
**

**Supplementary Figure 2 - Full length western blots from cropped images depicted in figure 3.** Samples from control or diabetic animals treated with the A_2A_R selective antagonist (S) or with the vehicle (V).


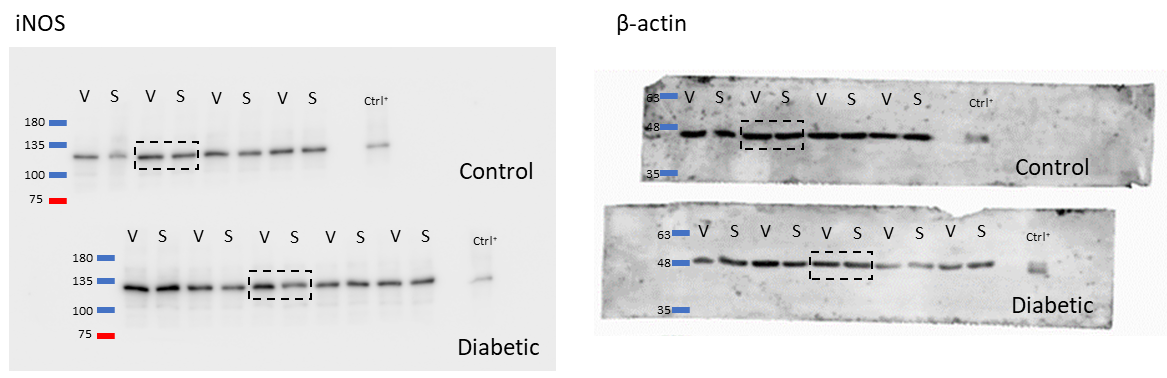


**Supplementary Figure 3 - Full length western blots from cropped images depicted in figure 4.** Samples from control or diabetic animals treated with the A_2A_R selective antagonist (S) or with the vehicle (V). Retinas from animals intravitreal injected with 2 µl of 1 mg/ml of lipopolysaccharide (LPS) were used as positive control.


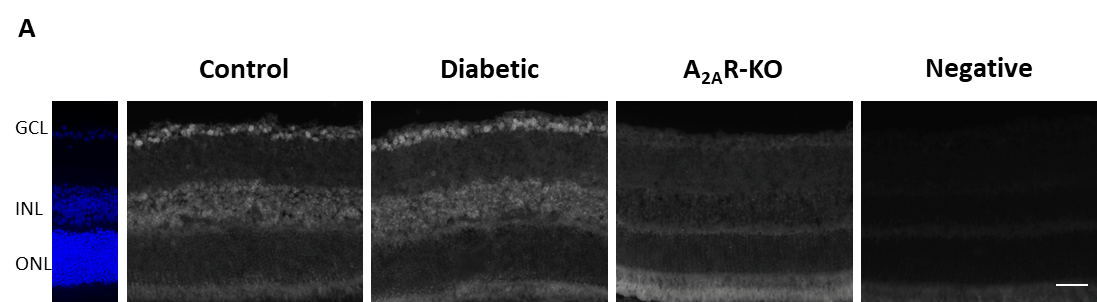


**Supplementary Figure 4 - Immunoreactivity of A_2A_R in mice retinal sections.** Retinal sections obtained from control and diabetic animals were immunolabelled for A_2A_R (antibody sc-13937, Santa Cruz Biotechnology). Retinal sections obtained from a mouse knockout for A_2A_R were also labelled against A_2A_R to confirm antibody specificity. Negative control was performed by omitting the primary antibody. Scale bar: 50 µm.
